# Mood disorders influencing endometriosis and adenomyosis: Mendelian randomisation study

**DOI:** 10.1192/bjo.2024.46

**Published:** 2024-04-16

**Authors:** Panyu Chen, Lei Jia, Cong Fang, Manchao Li

**Affiliations:** Department of Reproductive Medicine Center, Guangdong Engineering Technology Research Center of Fertility Preservation, Sixth Affiliated Hospital of Sun Yat-sen University, Guangzhou, Guangdong, China; and Biomedical Innovation Center, Sixth Affiliated Hospital, Sun Yat-sen University, Guangzhou, Guangdong, China

**Keywords:** Mood swings, major depressive disorder, endometriosis, adenomyosis, Mendelian randomisation study

## Abstract

**Background:**

Many studies have found an association between mood-disorder-related traits and endometriosis and adenomyosis. However, the cause–effect relationship remains unclear.

**Aims:**

We conducted Mendelian randomisation analyses to evaluate any causal relationship between mood disorders and endometriosis as well as different sites of endometriosis.

**Method:**

Summary-level statistics for mood-disorder-related traits and endometriosis (8288 cases, 68 969 controls) in European populations were derived from large-scale data-sets of genome-wide association studies. A two-sample Mendelian randomisation was performed using the inverse-variance weighted and weight median methods. Further sensitivity analyses, including heterogeneity, pleiotropy and leave-one-out analyses, were conducted to test the consistency of the results.

**Results:**

Genetically determined mood swings (odds ratio = 2.557, 95% CI: 1.192–5.483, *P* = 0.016) and major depression (odds ratio = 1.233, 95% CI: 1.019–1.493, *P* = 0.031) were causally associated with an increased risk of endometriosis. Mood swings (odds ratio = 4.238, 95% CI: 1.194–15.048, *P* = 0.025) and major depression (odds ratio = 1.512, 95% CI: 1.052–2.173, *P* = 0.025) were also causally associated with the risk of adenomyosis. Sensitivity analyses confirmed the reliability of the results.

**Conclusions:**

Our results suggest that mood-disorder-related traits increase the risk of endometriosis and adenomyosis. This study provides new insights into the potential pathogenesis of endometriosis and adenomyosis, and highlights the importance of preventing endometriosis and adenomyosis in patients with mood-disorder-related traits.

Endometriosis is defined as the presence of endometrial glands and stroma outside the uterine cavity.^1^ A frequent and common disease in women of reproductive age, it affects approximately 10% of women of reproductive age, according to the literature, accounting for more than 176 million women worldwide.^2^ Endometriosis is often associated with pelvic pain and infertility.^3,4^ As well as having a negative impact on the patient's quality of life, it is a major burden on the health resources of society.

The aetiology of endometriosis remains controversial. One of the most widely accepted theories is Sampson's retrograde menstruation theory, in which endometriosis arises as the result of retrograde flow of menstrual discharge from the uterus through the fallopian tubes, with spill of endometrial cells on to the ovary and other sites in the pelvis.^5^ Other pathogenic mechanisms include the coelomic metaplasia theory, which postulates that endometriosis originates from the metaplasia of specialised cells that are present in the mesothelial lining of the visceral and abdominal peritoneum;^6,7^ and the endometrial stem cells theory, which proposes that endometriosis arises from a single cell or a few specific cells with stem cell properties, including self-renewal and multi-lineage cell differentiation.^8^ Several studies have found endometriosis is often accompanied by a higher incidence of anxiety and depression.^9–11^ Cavaggioni et al confirmed that women with endometriosis were more likely to have dysfunctional disorders such as mood and anxiety disorders, autism, obsessive–compulsive disorder and depression than the general population.^12^ Research in recent years has provided new insights into the coexistence of psychological traits and endometriosis. A longitudinal study conducted in Sweden that lasted 17 years found that women with a history of affective disorders such as depression, anxiety and stress-related disorders, eating disorders, personality disorders, and attention-deficit hyperactivity disorder were more likely to be subsequently diagnosed with endometriosis.^13^ This opened up new speculation as to whether causal relationships exist between mood-disorder-related traits and endometriosis. Adenomyosis occurs when endometrial tissue grows into the muscular wall of the uterus. The mechanisms of adenomyosis include (a) invasion of endometrial basalis into the myometrium; (b) microtrauma of the junctional zone induced by tissue injury and repair; (c) *de novo* metaplasia from stem cells; and (d) outside-to-inside invasion induced by retrograde menstruation.

However, previous studies have failed to adequately elucidate the causal relationship between mood-disorder-related traits and endometriosis or adenomyosis, and the advent of Mendelian randomisation analyses provide a means of investigating causality. Therefore, we conducted two-sample Mendelian randomisation (TSMR) analyses to explore the causal effects of two mood-disorder-related traits (major depressive disorder and mood instability) on endometriosis and adenomyosis.

## Method

### Study design and data sources

TSMR was used as the primary analytic method, as it can overcome the main sources of bias resulting from classical observational epidemiology. The primary analyses aimed to explore the causality of two mood-disorder-related traits (mood instability and major depressive disorder) and endometriosis through a TSMR approach. The two representatives of exposure to ‘mood instability’ were (a) ‘mood swings’, as extracted from the UK Biobank, and (b) ‘major depression’, as extracted from the UK Biobank and Psychiatric Genomics Consortium.

We sought genome-wide association study (GWAS) summary data from studies with the largest available numbers of cases with European ancestry (to increase power and samples size), from the Medical Research Council Integrative Epidemiology Unit OpenGWAS project. We selected endometriosis, as well as different sites of endometriosis, including endometriosis of the uterus (known as ‘adenomyosis’ in this study), endometriosis of the ovary, endometriosis of the fallopian tube, endometriosis of the pelvic peritoneum, and endometriosis of the rectovaginal septum and vagina, as outcome variables. All original studies had obtained ethical approval and informed consent from the participants.

For instrumental variable selection, single-nucleotide polymorphisms (SNPs) associated with exposures with genome-wide significance (*P* < 5 × 10^−8^) were first selected. To avoid correlations between instrumental variables, we tested the linkage disequilibrium in selected SNPs. A clumping procedure was performed to screen independent SNPs with a threshold of 10 000 kb and *r*^2^ < 0.001. To avoid weak instruments, we also calculated F-statistics for all SNPs. If *F* values were greater than 10, we considered that there was no weak instrumental variable bias. The Mendelian randomisation between mood-disorder-related traits and endometriosis was principally analysed using the inverse variance weighted (IVW) and weight median methods; the Mendelian randomisation between mood-disorder-related traits and different sites of endometriosis was principally analysed using the IVW method; and causal estimates were converted to odds ratios (ORs) for readability purposes.

### Sensitivity analyses

Sensitivity analyses were conducted using the following methods. First, we performed Cochran's Q test to evaluate the heterogeneity among individual SNPs. A *P*-value greater than 0.05 was considered to indicate no heterogeneity. Potential outlier variants were detected by MR-PRESSO. Second, MR-Egger was used to test the horizontal pleiotropy of instrumental variables. If the *P*-value was greater than 0.05, the IVW estimate was considered to show no bias. Third, we conducted a leave-one-out sensitivity test to exclude the possibility that certain SNPs contributed to the results of this study. Last, the robust adjusted profile score (RAPS) was used to further determine causality and test for the presence of horizontal pleiotropy. All statistical analyses were carried out using the ‘TwoSampleMR’, ‘MR-PRESSO’ and ‘mr.raps’ packages in R version 4.2.2, and all *P*-values were two-sided.

### Ethical approval

This study obtained ethical approval from the ethics committee of the Reproductive Medicine Research Center of the Sixth Affiliated Hospital of Sun Yat-sen University. Written informed consent was obtained from all participants.

## Results

A total of 55 and 47 instrumental variables were extracted for the effects of mood swings and major depression on endometriosis, respectively. F-statistics of these SNPs were all greater than 10 to ensure the exclusion of bias from weak instrumental variables. (Supplementary Data Sheet 1 and 2 available at https://doi.org/10.1192/bjo.2024.46).

**Fig. 1:**
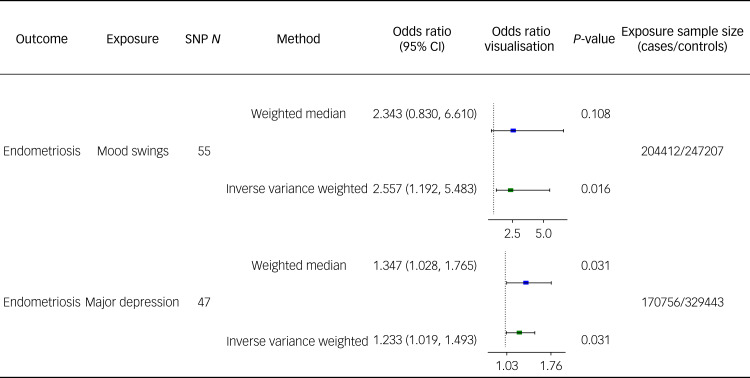
Forest plot of causality between mood disorders and endometriosis. SNP, single-nucleotide polymorphism.

Notably, the IVW method in the TSMR analyses showed a strong causal effect of both mood swings and major depression on endometriosis (odds ratio 2.557, 95% CI: 1.192–5.483; and odds ratio 1.233, 95% CI: 1.019–1.493, respectively, both *P* < 0.05). The odds ratio estimates from the weighted median analyses method also showed significant causal associations between major depression and endometriosis (odds ratio 1.347, 95% CI: 1.028–1.765, *P* < 0.05). The results the Mendelian randomisation analyses are shown as a forest plot in Fig. 1. RAPS analyses yielded effects similar to those of the IVW analysis (results shown in Supplementary Data Sheet 3).

**Fig. 2:**
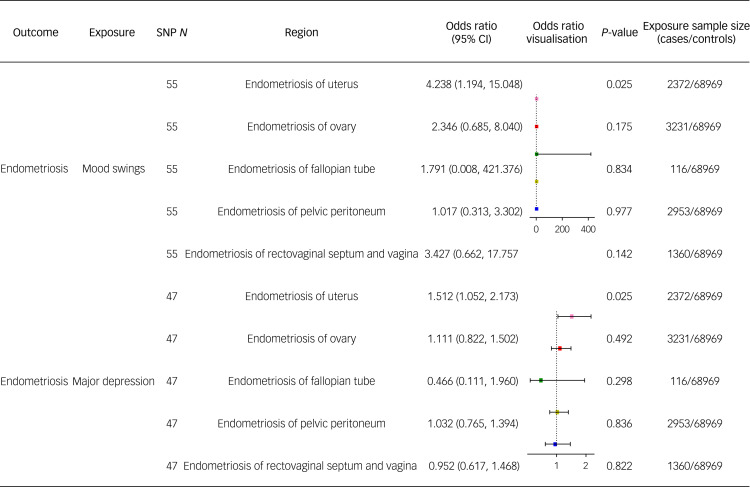
Forest plot of causality between mood disorders and different sites of endometriosis. SNP, single-nucleotide polymorphism.

The Mendelian randomisation analyses of the associations between mood-disorder-related traits and different sites of endometriosis showed strong causal effects of both mood swings and major depression on adenomyosis (odds ratio 4.238, 95% CI: 1.194–15.048; and odds ratio 1.512; 95% CI: 1.052–2.173, respectively, both *P* < 0.05). The odds ratio estimates obtained for mood swings, major depression, and the other sites of endometriosis (including ovary, fallopian tube, pelvic peritoneum, and rectovaginal septum and vagina) showed no significant associations. The results of the Mendelian randomisation analyses are shown as a forest plot in Fig. 2.

**Fig. 3:**
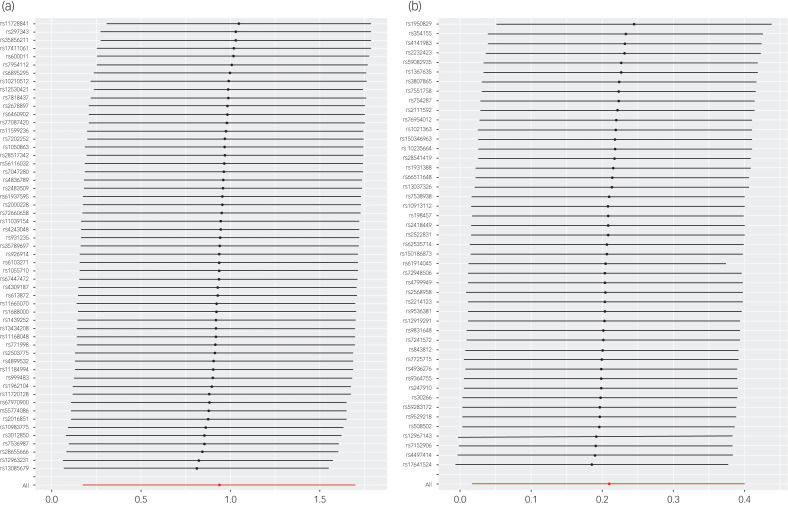
(a) Leave-one-out sensitivity analysis of the causal effect of mood swings on endometriosis. (b) Leave-one-out sensitivity analysis of the causal effect of major depression on endometriosis.

**Fig. 4:**
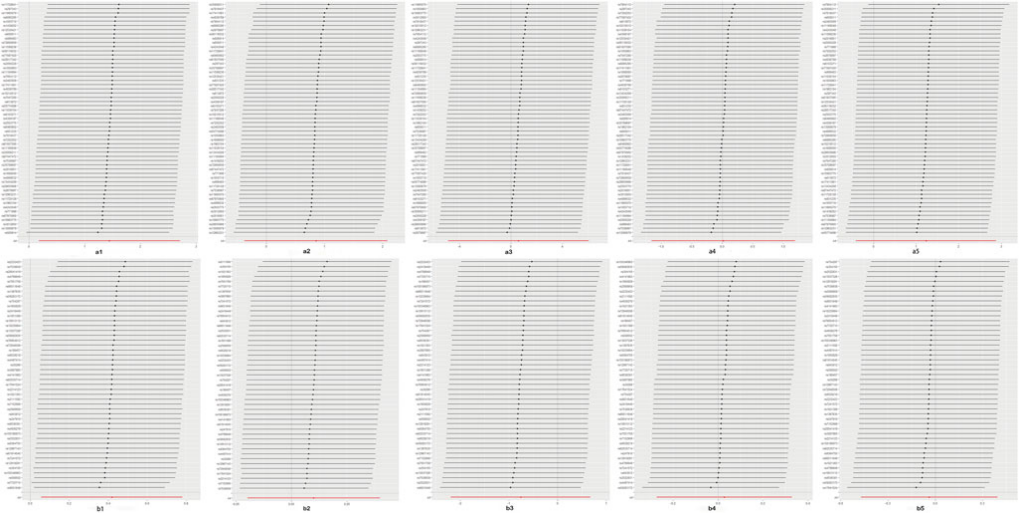
(a) Leave-one-out sensitivity analysis of the causal effects of mood swings on different sites of endometriosis. (b) Leave-one-out sensitivity analysis of the causal effects of mood swings on different sites of endometriosis. 1, uterus; 2, ovary; 3, fallopian tube; 4, pelvic peritoneum; 5, rectovaginal septum and vagina.

No evidence for directional pleiotropy was obtained when MR-Egger regression was performed (all *P* > 0.05), and MR-PRESSO analyses showed no outlier SNPs (Supplementary Data Sheet 4 and 5). There was no heterogeneity, as confirmed by both IVW and MR Egger analyses, according to Cochrane's Q test, with *P* greater than 0.05 (Supplementary Data Sheet 6). Leave-one-out plots suggested that the causal estimates were unlikely to be influenced by specific SNPs (Figs. 3 and 4). In addition, SNP effects individually and jointly from each Mendelian randomisation method are displayed in scatter plots (Supplementary Figure 1 and 2). No horizontal pleiotropy was detected, as the estimated overdispersion parameters calculated by RAPS were all very small (Supplementary Data Sheet 3).

## Discussion

This was the first Mendelian randomisation study to investigate the relationships of mood disorders with endometriosis and adenomyosis. Our results demonstrated a causal association between mood-disorder-related traits and endometriosis. Further analyses of studies focusing on different sites of endometriosis revealed a causal effect of mood-disorder-related traits on adenomyosis. Sensitivity analyses confirmed the reliability of the results.

In the past 20 years, several large epidemiological studies have found that endometriosis and symptoms related to mood disorders such as depression and anxiety may coexist.^9,14^ Both Cavaggioni et al and Li-Chi Chen et al reported that patients with endometriosis had a higher probability of suffering from affective-disorder-related symptoms than controls.^12,15^ Najjar's study confirmed that altered and dysregulated inflammatory cytokines are among the important pathogenic mechanisms of mood symptoms and/or disorders; this is also one of the pathogenic mechanisms of endometriosis.^16^ In recent years, a growing number of studies have found correlations between psychological traits and endometriosis. The results of a 17-year longitudinal study by Gao et al showed that all psychiatric disorders, except for non-affective psychotic disorders, were associated with increased prevalence of endometriosis.^13^ Adewuyi's study also provided evidence of a causal association between depression and endometriosis.^17^ This might have been due to the significant genetic overlap and correlation between these two traits.

However, observational studies cannot conclusively prove a causal association between mood-disorder-related traits and endometriosis because they cannot exclude the influence of confounding factors. Mendelian randomisation makes the exploration of causality in epidemiological studies possible by introducing instrumental variables that satisfy the core assumptions.^18^ Our study found for the first time that there is causal effect of mood swings and major depression on endometriosis, especially adenomyosis. This suggests that psychological factors may be among the potential pathogenic mechanisms of endometriosis. We propose a theory on the underlying mechanisms by which mood-disorder-related traits could cause endometriosis. Mood-disorder-related traits might shift the Th1/Th2 cytokine balance towards the Th2 response, which might lead to endometriosis. Chrousos et al found that psychological stress activated the hypothalamic–pituitary–adrenocortical axis and the sympathetic nervous system, leading to increased secretion of cortisol and catecholamines. Cortisol and catecholamines were able to suppress Th1 cytokines, thereby shifting the immune response toward a Th2 phenotype;^19^ this was also found in peritoneal fluid of patients with endometriosis by in a study by Olkowska-Truchanowicz.^20^ Another possible causal pathway involves abnormalities of the immune system. It is generally accepted that the key factors in the pathogenesis of endometriosis are immune dysfunction and inflammatory response at the site of the lesions,^21^ and there is growing evidence that immune dysregulation is one of the causes of psychiatric disorders.^22,23^ In accordance with this, a study by Cuevas et al found that stress may contribute to the development of endometriosis and exacerbate its severity in animal models through mechanisms that promote immunocyte recruitment, release of inflammatory mediators, and dysregulation of hippocampal hypothalamic–pituitary axis responses.^24^ Recent studies have suggested the possibility of a common genetic predisposition to both diseases, which is an alternative causality consistent with the results of our study. Koller's research showed that the coexistence of endometriosis with multiple psychiatric disorders is likely to be associated with genetic pleiotropy,^25^ and Li's study found that endometriosis induced pain sensitisation, anxiety and depression by modulating brain gene expression and electrophysiology.^26^

In the present study, the *F* values of all the SNPs were greater than 10; thus, the associations between SNPs and exposure factors were considered strong enough to satisfy the core hypothesis of the instrumental variables: the relevance assumption. Our sensitivity analyses found no evidence for directional or horizontal pleiotropy or heterogeneity, further confirming the reliability of the results.

In conclusion, our results indicate significant causal relationships between mood-disorder-related traits and endometriosis, especially adenomyosis. Our study thus provides new insights into the potential pathogenesis of endometriosis and adenomyosis and highlights the importance of preventing endometriosis and adenomyosis in patients with mood-disorder-related traits.

### Limitations

First, because the GWAS data in this study were only from a European population, it remains to be confirmed whether our findings can be applied to other populations; second, the exact mechanism by which mood-disorder-related traits cause endometriosis were not investigated in this study. In addition, it is not clear why mood-disorder-related traits were associated with adenomyosis but not other sites of endometriosis. The exact pathophysiological mechanism needs to be clarified.

### Implications for further research

As we found significant associations indicating a causal relationship between mood-disorder-related traits and endometriosis and adenomyosis, future research on the mechanisms of endometriosis and adenomyosis should include psychological factors. In the meantime, monitoring of diseases of the reproductive system, such as endometriosis and adenomyosis, in female patients with mood disorders should not be neglected.

## Supporting information

Chen et al. supplementary material 1 Chen et al. supplementary material

Chen et al. supplementary material 2 Chen et al. supplementary material

Chen et al. supplementary material 3 Chen et al. supplementary material

Chen et al. supplementary material 4 Chen et al. supplementary material

Chen et al. supplementary material 5 Chen et al. supplementary material

Chen et al. supplementary material 6 Chen et al. supplementary material

Chen et al. supplementary material 7 Chen et al. supplementary material

## Data Availability

GWAS summary statistics of mood-disorder-related traits and endometriosis are publicly available online (https://gwas.mrcieu.ac.uk/). The data that support the findings of this study are available within the article and its Supplementary files.
