# Supplementary figures and images for: Mood disorders influencing endometriosis and adenomyosis: Mendelian randomisation study
Source: BJPsych Open. 2024 Apr 16;10(3):e83. doi: 10.1192/bjo.2024.46 (PMC11060071; doi:10.1192/bjo.2024.46)

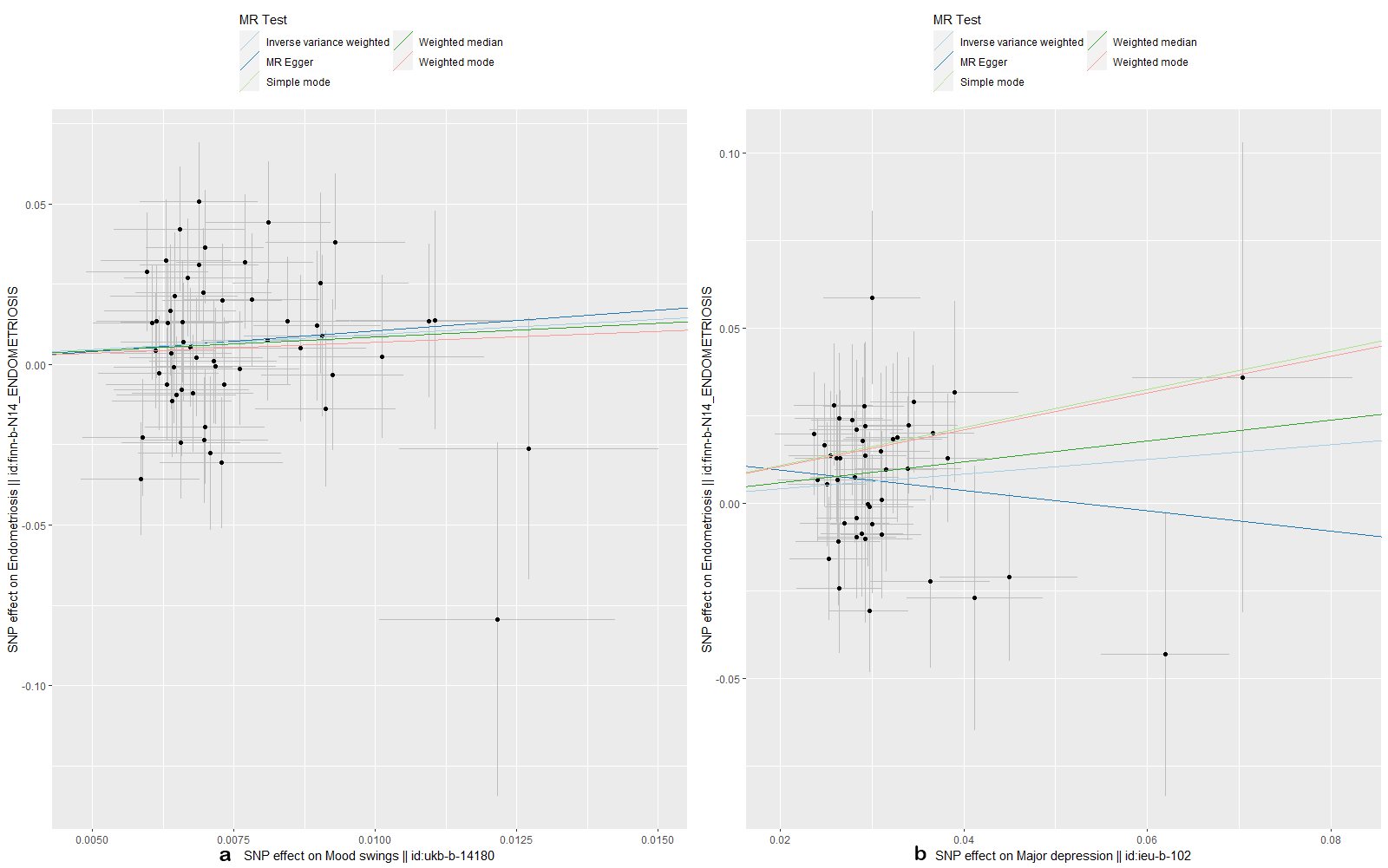

Supplement: Chen et al. supplementary material 7 — Chen et al. supplementary material [file S2056472424000462sup007.tif]
